# Pipkin type III femoral head fracture: which treatment strategy can be recommended?

**DOI:** 10.1186/s10195-023-00701-x

**Published:** 2023-06-16

**Authors:** Shanxi Wang, Xiaojun Yu, Bohua Li, Qing Ding, Tianqi Wang, Qin Li, Lei Liu, Hua Wu

**Affiliations:** 1 Department of Orthopedics, Tongji Hospital, Tongji Medical College, Huazhong University of Science and Technology, Jiefang Avenue 1095, Wuhan, 430030 People’s Republic of China; 2 Department of Orthopedics, West China Hospital, Sichuan University, 37# Guoxue Alley, Chengdu, 610041 Sichuan People’s Republic of China; 3 Department of Orthopaedics, The Eighth Affiliated Hospital, Sun Yat-sen University, Shenzhen, People’s Republic of China

**Keywords:** Femoral head fractures, Femoral neck fractures, Hip dislocation, Pipkin type III fractures

## Abstract

**Background:**

Pipkin type III femoral head fractures are relatively rare injuries. Few studies have explored and described the treatment and outcomes of Pipkin type III femoral head fractures. The purpose of this study was to evaluate the efficacy of open reduction and internal fixation (ORIF) in treating Pipkin type III femoral head fractures.

**Methods:**

We retrospectively reviewed 12 patients with Pipkin type III femoral head fractures who underwent ORIF from July 2010 and January 2018. The complications and reoperations were recorded. The visual analog scale (VAS) pain score, Harris hip score (HHS), Thompson–Epstein criteria, and SF-12 score [including the physical component summary (PCS) and the mental component summary (MCS)] were used for functional assessment.

**Results:**

Among the 12 patients, ten were males and two were females, with a mean age of 34.2 ± 11.9 years. The median follow-up time was 6 years (range 4–8 years). Five patients (42%) developed osteonecrosis of the femoral head, and one patient (8%) developed nonunion. These six patients (50%) underwent total hip arthroplasty (THA). One patient (8%) developed heterotopic ossification and underwent ectopic bone excision; this patient also presented with post-traumatic arthritis. The mean final VAS pain score and HHS were 4.1 ± 3.1 points and 62.8 ± 24.4 points, respectively. According to the Thompson–Epstein criteria, there was one patient (8%) with excellent, four patients (33%) with good, one patient (8%) with fair, and six patients (50%) with poor outcomes. The PCS score and MCS score were 41.7 ± 34.7 points and 63.2 ± 14.5 points, respectively.

**Conclusion:**

Limited by the high incidence of osteonecrosis of the femoral head, it is difficult to achieve satisfactory functional outcomes when treating Pipkin type III femoral head fractures using ORIF, and a primary THA may be considered. However, for younger patients, considering the survivorship of prosthesis, ORIF may be recommended with the proviso that the patient is fully informed of the high complication rate associated with this procedure.

Level of evidence: IV.

## Introduction

With the development of treatment concepts and surgical techniques, the management of femoral head fractures has evolved over the years [1–11]. Pipkin type III femoral head fractures, a subgroup of femoral head fractures, consist of femoral head fractures and ipsilateral femoral neck fractures [12]. Pipkin type III femoral head fractures account for approximately 8.6% of all femoral head fractures, making them the least common subtype of Pipkin fractures [4].

The prognosis of Pipkin type III femoral head fracture is worse than that of other subgroups of Pipkin fractures due to the severe damage to the blood supply to the femoral head [4, 10, 13]. However, due to its low incidence, only a few studies regarding the management of Pipkin type III femoral head fractures have been reported in the literature, and there is still no consensus on the treatment strategy for Pipkin type III femoral head fractures [1–3, 10, 12, 14–16]. As a result, it is difficult to choose an optimal treatment strategy when we encounter these injuries in clinical work.

Due to the severe damage to the femoral head blood supply, there is a significantly increased risk of femoral head necrosis in Pipkin type III femoral head fractures. Therefore, some authors have advocated total hip arthroplasty (THA) as the primary solution for Pipkin type III femoral head fractures [3, 13, 17–24]. However, femoral head fractures often occur in young people [4]. Considering the survivorship of prosthesis, we should be more prudent when choosing the treatment strategy, and open reduction and internal fixation (ORIF) should be recommended in order to preserve the joint, especially for young patients [4, 10, 12, 15, 16, 25–29]. In an effort to better evaluate the clinical outcomes of Pipkin type III femoral head fractures and choose the optimal treatment strategy, we report our experience with 12 patients who underwent ORIF.

## Materials and methods

### Patients and methods

We performed this retrospective study after obtaining approval from our institutional review board and consent from the patients. Between July 2010 and January 2018, 12 patients with a Pipkin’s type III femoral head fracture who underwent ORIF in our hospital were retrospectively reviewed. The patient’s age, gender, affected side, cause, injury severity score (ISS), time from injury to reduction of hip dislocation, and follow-up time were collected. The preoperative fracture assessment included anteroposterior pelvis radiographs, anteroposterior and lateral hip radiographs, and a CT scan of the pelvis. After the operation, a routine clinical follow-up was performed, and serial radiographs were obtained at every follow-up. To avoid examiner bias, postoperative evaluations were conducted by an independent surgeon not involved in the surgical treatment of these patients. All results are based on the radiographs and clinical records available.

### Surgical technique

All surgery was performed in our center by the same surgical team consisting of two senior orthopedic surgeons. Under general anesthesia, on a standard radiolucent table, the patient was placed in a lateral decubitus position. A Kocher–Langenbeck incision was performed, and the fascia lata was incised along the skin incision. After identifying the gluteus medius and minimus, the hip capsule was exposed through the abduction and internal rotation of the leg. The piriformis tendon was tagged and released approximately 1.5 cm from its insertion to protect the blood supply from the ascending branch of the medial circumflex femoral artery while the short external rotators were preserved, and the sciatic nerve was identified and protected. The short external rotators were gently pulled laterally by Hohmann retractors to protect the sciatic nerve and expose the surgical field. The hip was gently dislocated by flexion, adduction, and internal rotation, and a T-shaped capsulotomy was performed to expose the femoral head fracture. Small or comminuted fragments were excised. The large fragments of femoral head were reduced anatomically and fixed with bioabsorbable screws or cannulated screws. All screw heads were countersunk below the cartilage level. Next, the femoral neck fracture was reduced and fixed with three cannulated screws, and the hip was reduced gently. Finally, the articular capsule and piriformis tendon were repaired, and the wound was closed in layers after the placement of a drain.

### Postoperative management

Prophylactic intravenous antibiotics were administered for 24 h postoperatively, and low molecular weight heparin was given to prevent deep venous thrombosis. The drain was removed within 24 h after the operation. All patients were instructed to perform functional exercises of the quadriceps femoris on the second day after operation, and no weight bearing or only toe-touch weight bearing was required for 6–8 weeks initially. After discharge, routine clinical follow-ups were conducted monthly until radiographic fracture healing was achieved, and then annually. Fracture healing was identified on an X-ray or CT scan as the presence of a blurred fracture line with continuous trabeculae. Once the radiographs showed fracture healing, progressive weight bearing was started.

### Outcome measures

The primary outcome measures were complications and reoperations. The complications included wound infection, deep venous thrombosis, nonunion, post-traumatic osteoarthritis, osteonecrosis of the femoral head, and heterotopic ossification.

The secondary outcome measure was final functional outcomes. To investigate this, patient-reported outcome measures including the visual analog scale (VAS) pain score, Harris hip score (HHS), Thompson–Epstein criteria, and SF-12 score [including the physical component summary (PCS) and the mental component summary (MCS)] were evaluated at the final follow-up [30–33]. Patients who underwent additional THA were classified as having poor functional outcomes regardless of the final hip functional outcomes, and the preoperative functional evaluation was adopted as the final functional outcomes.

### Statistical analysis

The Statistical Package for the Social Sciences version 20.0 (SPSS version 20.0, IBM Corp.) was used for the statistical analysis. Normality was tested using the Kolmogorov–Smirnov test. Continuous variables with normal distributions were expressed as the mean ± standard deviation (SD); other continuous variables were expressed as the median and range. Categorical variables were expressed as the number and percentage. Independent* t*-tests were used for normally distributed continuous data, and the Mann–Whitney test was used to compare abnormally distributed continuous data between two groups. The chi-square test or Fisher’s exact test was used to analyze the categorical variables. The level of significance was set at *p* < 0.05.

## Results

### General data

**Fig. 1:**
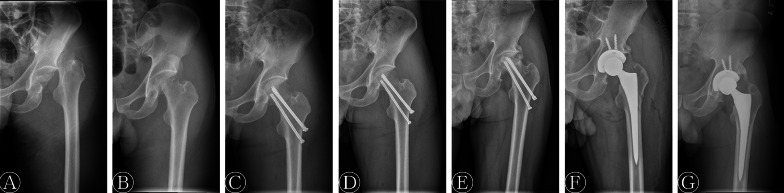
A 48-year-old man with a left Pipkin type III femoral head fracture. **A** Radiograph after injury. **B** Radiograph after attempted closed reduction of posterior hip dislocation. **C** Radiograph after open reduction and internal fixation. **D** Radiograph at 3 months postoperatively showing bony union. **E** Radiograph at 36 months postoperatively showing osteonecrosis of the femoral head and heterotopic ossification. **F** Radiograph after total hip arthroplasty. **G** Radiograph at final follow-up

Among the 12 patients, ten patients (83%) were males and two patients (17%) were females. The mean age at the time of fracture was 34.2 ± 11.9 years. The right hip was affected in five patients (42%) and the left hip was affected in seven patients (58%). The mean BMI was 23.4 ± 1.5 kg/m^2^. Nine patients (75%) sustained the injury in a traffic accident, two patients (17%) fell from a height, and one patient (8%) sustained a bruise injury. The mean ISS was 14.8 ± 5.3 points. Prompt closed or open reduction of hip dislocation was attempted under general anesthesia in all patients (Fig. 1). Seven patients (58%) were reduced within 6 h, whereas the dislocation of the hip was reduced within 6 to 12 h in five patients (42%), and the mean time from injury to the reduction of the hip was 7.1 ± 3.1 h. Two patients (17%) underwent excision of the femoral head fragments, and ten patients (83%) underwent ORIF of the femoral head fragments. The median follow-up time was 6 years (range 4–8 years) (Table 1).

**Table 1:** Patient demographics and clinical data

Patient no.	Gender	Age (years)	Side	BMI (kg/m^2^)	Cause	ISS	HR time (h)	Treatment (FHF)	Follow-up (years)
1	Male	30	Left	24.8	Traffic accident	14	5	ORIF	7
2	Male	45	Right	23.7	Traffic accident	14	6	ORIF	8
3	Male	27	Left	22.2	Traffic accident	14	5	ORIF	7
4	Male	35	Right	22.3	Traffic accident	10	4	ORIF	6
5	Female	28	Right	22.5	Falling from a height	10	3	ORIF	4
6	Male	24	Left	21.3	Traffic accident	27	6	Excision	7
7	Male	21	Right	22.2	Traffic accident	10	10	Excision	6
8	Male	52	Left	26.4	Bruise injury	19	9.5	ORIF	7
9	Female	52	Left	25.0	Traffic accident	10	9	ORIF	6
10	Male	21	Left	22.6	Falling from a height	19	11.5	ORIF	5
11	Male	48	Left	24.3	Traffic accident	11	12	ORIF	6
12	Male	27	Right	23.2	Traffic accident	19	4	ORIF	4

*ISS* injury severity score, *HR* hip reduction, *FHF* femoral head fragments, *ORIF* open reduction and internal fixation

### Complications, reoperation, and functional outcomes

**Fig. 2:**
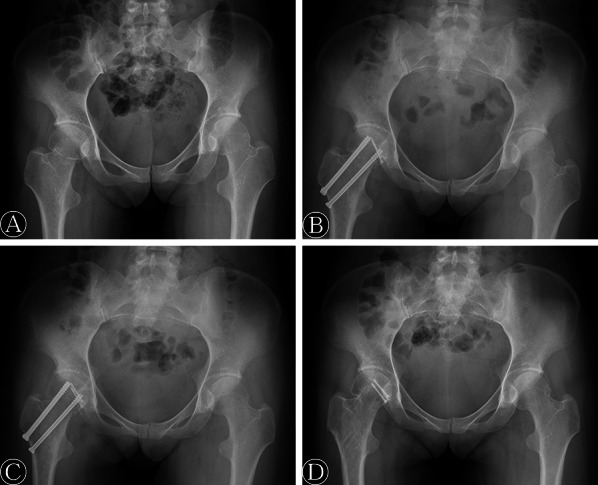
A 28-year-old woman with a right Pipkin type III femoral head fracture. **A** Preoperative anteroposterior radiograph of the pelvis. **B** Anteroposterior radiograph of the pelvis after open reduction and internal fixation. **C** Anteroposterior radiograph of the pelvis at 3 months postoperatively, demonstrating bony union. **D** Radiograph at 46 months after surgery

Seven of twelve patients (58%) experienced major complications and reoperation. One patient (8%) developed a superficial wound infection which was cured by performing dressing changes and using an antibiotic treatment. No deep venous thrombosis was found in this series. Five patients (42%) developed osteonecrosis of the femoral head, and these five patients underwent an additional THA. One patient (8%) suffered from nonunion of the femoral neck fracture and internal fixation failure. This patient was converted to THA 5 months after the initial operation. One patient (8%) developed heterotopic ossification (Brooker type IV) and underwent ectopic bone excision at 18 months after the initial operation because of the pain and the limitation on hip motion. This patient had developed heterotopic ossification (Brooker type II) again at the final follow-up, and the patient also presented with post-traumatic arthritis. The mean final VAS pain score and Harris hip score were 4.1 ± 3.1 points and 62.8 ± 24.4 points, respectively. According to the Thompson–Epstein criteria, there was one patient (8%) with excellent (Fig. 2), four patients (33%) with good, one patient (8%) with fair, and six patients (50%) with poor outcomes. For the final health status, the PCS score and MCS score were 41.7 ± 34.7 points and 63.2 ± 14.5 points, respectively (Table 2).

**Table 2:** Patient outcome measures

Patient no.	VAS pain score	Harris score	T-E criteria	SF-12 score		Complications	Additional surgery
				PCS	MCS		
1	1	86	Good	70	75.0	None	None
2	1	83	Good	75	70.8	None	None
3	7	41	Poor	0	45.8	ONFH	THA
4	6	44	Poor	10	50.0	ONFH	THA
5	0	95	Excellent	95	91.7	None	None
6	7	44	Poor	10	62.5	ONFH	THA
7	0	90	Good	75	75.0	None	None
8	4	73	Fair	45	62.5	HO, PTO	EBE
9	6	47	Poor	20	50.0	ONFH	THA
10	7	44	Poor	25	54.2	Nonunion	THA
11	8	24	Poor	0	45.8	ONFH	THA
12	2	83	Good	75	75.0	None	None

*VAS* visual analog scale, *T-E criteria* Thompson–Epstein criteria, *SF-12* short form 12-item questionnaire score, *PCS* physical component summary, *MCS* mental component summary, *ONFH* osteonecrosis of the femoral head, *HO* heterotopic ossification, *PTO* post-traumatic osteoarthritis, *THA* total hip arthroplasty, *EBE* ectopic bone excision

## Discussion

Femoral head fractures are not common and are usually secondary to posterior dislocation of the hip [5, 12, 17, 34]. In 1957, Pipkin classified this injury into four subtypes, of which type III fractures are the least common. They are characterized by femoral head fracture and ipsilateral femoral neck fracture [12]. The mechanism of Pipkin type III femoral head fractures is described as the application of two forces to the hip joint. The first axial force causes the hip dislocation and femoral head fracture, then the second force after dislocation shears the femoral head against the iliac wing and causes femoral neck fracture [11, 12].

Few studies have explored and described the treatment and outcomes of Pipkin type III femoral fracture, and the optimal treatment strategy remains controversial. Due to the severe compromise of the femoral head blood supply, there is a significantly increased risk of head necrosis in Pipkin type III femoral head fractures [1, 3, 16, 20]. Scolaro et al. [1] reported that among seven patients with Pipkin type III femoral head fractures who were initially treated with ORIF, six patients developed osteonecrosis of the femoral head. Tonetti et al. [22] reported four cases of Pipkin type III femoral head fracture; three patients treated with ORIF developed osteonecrosis of the femoral head and underwent an additional THA. In another study, Park et al. [14] reported five iatrogenic Pipkin type III femoral head fractures; three of the patients were treated with arthroplasty while two patients were treated with ORIF. However, the two patients treated with ORIF suffered nonunion and osteonecrosis of the femoral head during follow-up and required total hip arthroplasty. Therefore, more and more authors have advocated THA as the primary solution for Pipkin type III femoral head fractures [3, 13, 17–24]. In our series, we retrospectively reviewed 12 patients with Pipkin’s type III femoral head fracture who were initial treated with ORIF and found that 58% of the patients experienced major complications and reoperations. Among them, one patient presented nonunion and five patients developed osteonecrosis of the femoral head. All six of these patients underwent an additional THA. The mean time from primary operation to additional THA was 27.7 months. Considering that the outcome of THA after prior ORIF surgeries is suboptimal compared to the outcome of direct primary THA [35, 36], initial THA might be the optimal treatment option when a Pipkin type III femoral head fracture is encountered.

However, femoral head fractures often occur in young people, with the average age of injury being 38.9 years [4]. Based on previous reports in the literature, the 20-year prosthesis survivorship after primary THA in patients under 35 years ranges from 41 to 66%, and there is currently limited evidence on the use of THA for primary treatment of hip fractures in young individuals [37–39]. Consequently, we should choose the treatment strategy used in young patients with Pipkin type III femoral head fractures more prudently, and ORIF should be recommended to preserve the joint, while joint arthroplasty is more suitable for elderly patients [4, 10–12, 15, 16, 25–29].

When ORIF is chosen as the initial treatment, several important factors need to be considered. The first is the time from injury to reduction of the hip dislocation. Prolonged hip dislocation can cause a vasospasm and a progressive increase in intracapsular pressure, resulting in further damage to the blood supply of the femoral head [40]. Early and prompt hip reduction is beneficial for obtaining a better functional outcome [4, 6, 41, 42]. Our previous work showed that urgent reduction of the hip dislocation within 6 h is associated with a lower incidence of osteonecrosis of the femoral head compared to reduction of hip dislocation over 6 h (5.1% vs. 15.6%) [42]. However, considering the rarity of the Pipkin type III femoral fracture—only 12 patients were enrolled in this study—it is impossible to perform a powerful subgroup analysis, so future systematic reviews may be required to further explore the effect of hip reduction time on the functional outcomes of Pipkin type III fractures treated by ORIF.

Next, in this study, all patients were treated through the posterior Kocher–Langenbeck approach. Compared with other surgical approaches, the posterior approach is associated with an increased incidence of osteonecrosis of the femoral head [11, 43, 44]. Although the anterior approach causes less damage to the blood supply to the femoral head, the increased risk of HO and poor exposure of the posterior structure limits its applicability, and it is more suitable for Pipkin type I and II femoral head fractures [14, 15]. Besides, it is difficult to reduce a femoral head fracture in a non-reducible femoral head fracture-dislocation through an anterior approach [7, 14]. Ganz et al. [45] described a posterior-based approach involving surgical dislocation combined with trochanteric-flip osteotomy. This approach can avoid damaging the deep branch of the medial femoral circumflex artery and provide good exposure of the femoral head [11, 45, 46]. More importantly, it is reported that the Ganz approach is associated with a lower incidence of osteonecrosis of the femoral head [5, 45, 46]. Although there is a risk of nonunion after trochanteric osteotomy [45, 46], the Ganz approach may be a better choice once the initial ORIF has been chosen for treating the Pipkin type III femoral head fracture.

In addition, anatomic reduction of fractures is imperative for a satisfactory prognosis, and poor reduction of the fracture is associated with a higher rate of post-traumatic osteoarthritis [1, 4, 5, 10]. The management of femoral head fragments is determined by multiple factors, including size, location, and degree of comminution [5]. In general, fragments that are less than 1 cm in size or located in the non-weight-bearing area can be excised; otherwise, anatomic reduction and internal fixation should be used [11].

Like other retrospective case series, this study has several limitations. Chief among these limitations is the small sample size, which makes it impossible to perform a powerful subgroup analysis based on relevant factors such as age and interval between injury and hip reduction. Future systematic reviews may be required to determine the age limit for choosing ORIF or THA as the initial treatment for type III fractures. Second, the follow-up time is insufficient, and we cannot assess the long-term outcomes of ORIF when it is used to treat Pipkin type III femoral head fractures. Furthermore, in this study, all patients were treated by ORIF, so there may have been a selection bias. A comparative study with ORIF versus direct THA and a long-term follow-up of at least 10 years is needed to evaluate the efficacies of the two methods in treating Pipkin type III femoral head fractures.

## Conclusion

Limited by the high incidence of osteonecrosis of the femoral head, it is difficult to obtain a satisfactory prognosis and satisfactory functional outcomes of ORIF as an initial surgical method for the treatment of Pipkin type III femoral head fractures, and a primary THA may be considered. However, for younger patients, considering the survivorship of prosthesis, ORIF may be recommended with the proviso that the patient is fully informed of the high complication rate associated with this procedure.

## Data Availability

The datasets used and analyzed during the current study are available from the corresponding author on reasonable request.
